# Putative Novel miR-1133 Is Associated with Oxidative Stress in Hyperglycemia-Induced Endothelial Cells

**DOI:** 10.3390/biomedicines14092038

**Published:** 2026-09-10

**Authors:** Nur Syakirah Othman, Amilia Aminuddin, Adila A. Hamid, Shahidee Zainal Abidin, Saiful Effendi Syafruddin, Mohd Faizal Ahmad, Farah Hanan Fathihah Jaffar, Nur Athirah Othman Basri, Azizah Ugusman

**Affiliations:** 1 Department of Physiology, Faculty of Medicine, Universiti Kebangsaan Malaysia, Kuala Lumpur 56000, Malaysia; dr.nursyakirahothman@gmail.com (N.S.O.); amilia@hctm.ukm.edu.my (A.A.); adilahamid@ukm.edu.my (A.A.H.); p131665@siswa.ukm.edu.my (N.A.O.B.); 2 Cardiovascular and Pulmonary (CardioResp) Research Group, Universiti Kebangsaan Malaysia, Bangi 43600, Malaysia; 3 Faculty of Science and Marine Environment, Universiti Malaysia Terengganu, Kuala Nerus 21030, Malaysia; shahidee.zainal@umt.edu.my; 4 UKM Medical Molecular Biology Institute (UMBI), Universiti Kebangsaan Malaysia, Kuala Lumpur 56000, Malaysia; effendisy@hctm.ukm.edu.my; 5 Department of Obstetrics and Gynecology, Faculty of Medicine, Universiti Kebangsaan Malaysia, Kuala Lumpur 56000, Malaysia; drmohdfaizal@ukm.edu.my; 6 Faculty of Health Sciences, MAIWP International University, Kuala Lumpur 68100, Malaysia

**Keywords:** diabetes mellitus, endothelial dysfunction, hyperglycemia, putative novel miR-1133, oxidative stress, PIK3CA

## Abstract

**Background:** Diabetes mellitus is characterized by chronic hyperglycemia, which promotes oxidative stress and endothelial dysfunction. MicroRNAs (miRNAs) have emerged as important regulators of diabetic vascular complications. Our previous RNA-sequencing study identified putative novel miR-1133 as an upregulated miRNA in hyperglycemia-induced human umbilical vein endothelial cells (HUVECs). This study aimed to investigate the potential involvement of putative novel miR-1133 in oxidative stress in hyperglycemia-induced HUVECs. **Methods:** Functional enrichment and protein–protein interaction (PPI) network analyses were performed to identify biologically relevant predicted target genes of putative novel miR-1133. Phosphatidylinositol-4,5-bisphosphate 3-kinase catalytic subunit alpha (*PIK3CA*) was selected for further investigation because it was identified as a hub gene in the PPI network and was involved in the enriched PI3K/Akt signaling pathway. HUVECs were exposed to high glucose (33.3 mM) to establish an in vitro hyperglycemic model, and the effects of transfection with putative novel miR-1133 inhibitor on *PIK3CA* expression and oxidative stress markers were evaluated. **Results:** Bioinformatic analyses identified *PIK3CA* as a hub gene among the predicted targets of putative novel miR-1133 and identified the PI3K/Akt signaling pathway as significantly enriched. Experimentally, hyperglycemia significantly reduced *PIK3CA* expression, whereas putative novel miR-1133 inhibitor transfection increased *PIK3CA* expression. Furthermore, putative novel miR-1133 inhibitor transfection was associated with reduced reactive oxygen species and 8-hydroxy-2′-deoxyguanosine levels, together with increased superoxide dismutase 1 (*SOD1*) mRNA expression and total SOD activity. **Conclusions:** Transfection with putative novel miR-1133 inhibitor was associated with increased *PIK3CA* expression and reduced oxidative stress markers in hyperglycemia-induced endothelial cells. Together, bioinformatic and experimental findings support an association between putative novel miR-1133 inhibitor transfection, *PIK3CA* expression, and oxidative stress under hyperglycemic conditions. Further studies are required to validate the direct interaction between putative novel miR-1133 and *PIK3CA* and to determine the relevance of these findings in vivo.

## 1. Introduction

Cardiovascular disease is the leading cause of mortality and morbidity among individuals with diabetes mellitus (DM). Globally, the prevalence of DM is projected to increase from 536 million cases in 2021 to 783 million by 2045, representing a major public health concern [1]. Uncontrolled DM can lead to macrovascular complications, including cerebrovascular disease, coronary artery disease and peripheral vascular disease, as well as microvascular complications such as diabetic neuropathy, retinopathy, and nephropathy [2]. Endothelial dysfunction, primarily triggered by chronic hyperglycemia, is a key mechanism underlying diabetic vascular complications [3].

Hyperglycemia enhances the generation of reactive oxygen species (ROS), resulting in oxidative stress and endothelial dysfunction [4]. Endothelial dysfunction is characterized by reduced nitric oxide (NO) bioavailability, increased vascular tone, inflammation, excessive ROS production, and platelet aggregation, all of which contribute to atherogenesis and diabetic vascular complications [5]. Although ROS play important roles as secondary messengers in intracellular signaling pathways, excessive ROS production disrupts cellular redox balance and damages lipids, proteins, and deoxyribonucleic acid (DNA), ultimately leading to cellular dysfunction [6].

To counteract oxidative stress, cells activate endogenous antioxidant defense mechanisms [7]. Superoxide dismutase (SOD) is a key antioxidant enzyme that converts superoxide radicals into oxygen and hydrogen peroxide, serving as an important first-line defense against oxidative damage [8]. In addition, 8-hydroxy-2-deoxyguanosine (8-OHdG), an oxidized derivative of deoxyguanosine generated following ROS-induced DNA damage, is widely used as a biomarker of oxidative stress [9]. Elevated levels of 8-OHdG have been reported in oxidative stress-related diseases, including diabetes [10], atherosclerosis [11], and stroke [12].

In recent years, microRNAs (miRNAs) have emerged as important regulators in the pathogenesis of diabetic vascular complications [13,14]. miRNAs are small non-coding RNAs that regulate gene expression at the post-transcriptional level through sequence-specific interactions with target messenger RNAs (mRNAs) [15]. Several annotated miRNAs, including miR-21 and members of the miR-200 family, have been implicated in oxidative stress and endothelial dysfunction under diabetic or high-glucose conditions [16,17,18]. However, advances in next-generation sequencing continue to identify previously uncharacterized miRNAs whose biological functions remain largely unknown, particularly in diabetic vascular disease.

In our previous small RNA sequencing study, we identified a putative novel miRNA, designated novel miR-1133, as one of the most highly upregulated miRNAs in hyperglycemia-induced human umbilical vein endothelial cells (HUVECs) [19]. This putative miRNA was predicted using the miRDeep2 algorithm following alignment of sequencing reads to the human reference genome and comparison with known miRNAs in miRBase and was subsequently validated by stem-loop quantitative reverse transcription polymerase chain reaction (RT-qPCR) [19]. The designation “novel miR-1133” was an internal identifier automatically assigned by the miRDeep2 prediction pipeline and was retained for consistency with our previous publication. At the time of this study, this putative miRNA had not been annotated in public miRNA repositories and does not represent an official miRBase-approved miRNA nomenclature. Therefore, throughout this article, novel miR-1133 refers to a putative novel miRNA identified in our previous study [19] rather than an established annotated human miRNA.

In the same study, miRDB target prediction identified 132 potential target genes of putative novel miR-1133, which were reported in the Supplementary Materials [19]. However, the functional enrichment and network analyses in that study were conducted in the broader context of the differentially expressed miRNAs rather than specifically focusing on the 132 predicted targets of putative novel miR-1133. Thus, the biological significance and potential downstream targets of putative novel miR-1133 remained to be investigated.

In the present study, Gene Ontology (GO), Kyoto Encyclopedia of Genes and Genomes (KEGG), and protein–protein interaction (PPI) network analyses were specifically performed on the 132 previously predicted target genes of putative novel miR-1133. These analyses identified several biological pathways, including the phosphatidylinositol signaling pathway, and identified phosphatidylinositol-4,5-bisphosphate 3-kinase catalytic subunit alpha (*PIK3CA*) as a major hub gene within the predicted regulatory network. Given the established role of phosphatidylinositol 3-kinase (PI3K) signaling in endothelial survival, redox homeostasis, nitric oxide production, and vascular function [20,21,22], *PIK3CA* was selected as a biologically plausible candidate gene for further experimental investigation. However, these bioinformatic findings were predictive, and the association of putative novel miR-1133 with oxidative stress and *PIK3CA* expression had not been experimentally investigated.

Therefore, the present study aimed to investigate the association of putative novel miR-1133 with *PIK3CA* expression and oxidative stress in hyperglycemia-induced endothelial cells. Specifically, we examined whether transfection with putative novel miR-1133 inhibitor was associated with changes in *PIK3CA* expression and oxidative stress markers, including intracellular ROS, superoxide dismutase 1 (*SOD1*) expression, total SOD activity, and 8-OHdG levels, in hyperglycemia-induced HUVECs. Rather than establishing a direct miRNA–target interaction or a causal *PIK3CA*-dependent mechanism, the present study evaluates these associations and provides a basis for future mechanistic studies.

## 2. Materials and Methods

### 2.1. Human Umbilical Vein Endothelial Cells (HUVECs) Isolation and Culture

Umbilical cords were obtained from healthy, term pregnancies without known metabolic or vascular complications. HUVECs were isolated from freshly obtained human umbilical cords using an enzymatic digestion method, as previously described [23]. Briefly, umbilical veins were perfused with 0.01% (*w*/*v*) collagenase Type I (Worthington Biochemical Corporation, Lakewood, NJ, USA) to detach endothelial cells. Cell identity was confirmed by the characteristic cobblestone morphology together with positive expression of CD31 and von Willebrand factor. HUVECs were cultured in endothelial cell medium (ScienCell Research Laboratories, Carlsbad, CA, USA) supplemented with 5% fetal bovine serum, 1% endothelial cell growth supplement and 1% penicillin–streptomycin, and maintained at 37 °C in a humidified incubator containing 5% CO_2_. The culture medium was replaced every two days until the cell monolayer became confluent. All experiments were conducted using primary HUVECs at passages 3–5 when cultures reached approximately 80% confluence.

### 2.2. Hyperglycemia Induction

To establish an in vitro hyperglycemic HUVEC model, HUVECs were cultured in endothelial cell medium containing 33.3 mM D-glucose (Sigma-Aldrich, St. Louis, MO, USA) for 24 h, as previously described [19]. HUVECs cultured in endothelial cell medium containing 5.5 mM glucose served as the normal glucose control group. The glucose concentration (33.3 mM) and treatment duration (24 h) were selected to maintain consistency with our previous RNA-sequencing study, in which identical experimental conditions were used to identify putative novel miR-1133 in hyperglycemia-induced HUVECs [19]. These conditions were therefore adopted in the present study to investigate putative novel miR-1133 using the same experimental model.

### 2.3. Bioinformatic Analysis of Predicted Putative Novel miR-1133 Target Genes

Predicted target genes of putative novel miR-1133 were obtained from our previously published RNA-sequencing study, in which 132 potential target genes of putative novel miR-1133 in hyperglycemia-induced HUVECs were predicted using the miRDB target prediction database (http://mirdb.org) [19] (Supplementary Table S1). Functional enrichment analyses, including GO and KEGG pathway analyses, were performed using the Search Tool for the Retrieval of Interacting Genes/Proteins (STRING) database (version 12.0; http://string-db.org, accessed on 18 December 2024). The enrichment analyses were used to identify significantly enriched biological processes and signaling pathways associated with the predicted target genes.

A PPI network was subsequently constructed using the STRING database and visualized using Cytoscape software (version 3.10.3). Hub genes within the network were identified using the CytoHubba plugin based on degree centrality, and the top 10 hub genes with the highest interaction scores were selected for further evaluation. Among the identified hub genes, *PIK3CA* was selected for further experimental investigation based on its central position within the PPI network, the significant enrichment of the PI3K/Akt signaling pathway in the functional enrichment analysis, and its well-established role in endothelial cell function and oxidative stress regulation [20,21,22]. The bioinformatic analyses were used to generate hypotheses regarding the potential biological functions and downstream targets of putative novel miR-1133, which were subsequently evaluated through experimental studies.

### 2.4. Transient Transfection of Putative Novel miR-1133 Inhibitor

Following 24 h of hyperglycemia induction, HUVECs were transiently transfected with a custom-designed mirVana™ miRNA inhibitor targeting the mature putative novel miR-1133 sequence (5′-GCUGGGCGGCUUGCUGGGGCUCG-3′) (Thermo Fisher Scientific, Waltham, MA, USA) at a final concentration of 5 nM using Lipofectamine^®^ RNAiMAX transfection reagent (Thermo Fisher Scientific Inc., Waltham, MA, USA) according to the manufacturer’s instructions. Cells were maintained under hyperglycemic conditions (33.3 mM glucose) throughout the subsequent 24-h transfection period. HUVECs treated with Lipofectamine^®^ RNAiMAX alone without the putative novel miR-1133 inhibitor underwent the identical transfection procedure and served as the transfection control to account for any nonspecific effects associated with the transfection reagent and procedure. The total experimental duration for the transfection experiment was 48 h, comprising a 24-h hyperglycemic induction followed by a 24-h transfection period under continued hyperglycemic conditions. Cells were harvested immediately after transfection for subsequent analyses.

Prior to the experimental studies, the transient transfection protocol was validated using the commercially available positive control miRNA inhibitor hsa-let-7c (Thermo Fisher Scientific, Waltham, MA, USA). Successful delivery of a biologically active miRNA inhibitor was confirmed by the expected increase in expression of its known target gene, *HMGA2*, demonstrating successful implementation of the transfection protocol (Supplementary Figure S1). Putative novel miR-1133 expression was not directly quantified following inhibitor transfection in the present experiments.

### 2.5. Quantitative Real-Time Polymerase Chain Reaction (qPCR)

Total RNA was extracted from HUVECs using the miRNeasy Kit (Qiagen, Germantown, MD, USA) according to the manufacturer’s instructions. Complementary DNA (cDNA) was synthesized using the M-MLV Reverse Transcriptase First-Strand cDNA Synthesis Kit (Biosearch Technologies, Hoddesdon, UK). *PIK3CA* mRNA expression was quantified using the commercial *PIK3CA* primer (QT00014861; Qiagen, USA) according to the manufacturer’s instructions. For superoxide dismutase 1 *(SOD1)* gene expression analysis, custom primers were designed using Primer3 software based on sequences retrieved from the NCBI GenBank database. *GAPDH* was used as the endogenous reference gene for *SOD1* quantification, and the primer sequences were as follows: *SOD1* forward, 5′-GTTGCAGTCCTCGGAACCAG-3′; *SOD1* reverse, 5′-CCACACCTTCACTGGTCCAT-3′; *GAPDH* forward, 5′-ACAGTTGCCATGTAGACC-3′; and *GAPDH* reverse, 5′-TTGAGCACAGGGTACTTTA-3′. The selection of *GAPDH* was supported by previous studies demonstrating stable *GAPDH* expression in HUVECs under hyperglycemic conditions and its use as a reference gene in high-glucose HUVEC models [24,25,26]. However, *GAPDH* stability was not independently evaluated under the specific experimental conditions of the present study.

Quantitative PCR was performed using qPCRBIO SyGreen^®^ Mix (PCR Biosystems, London, UK) on a Bio-Rad CFX96 Real-Time PCR System (Bio-Rad Laboratories, Hercules, CA, USA). The thermal cycling conditions consisted of initial denaturation at 95 °C for 2 min, followed by 40 cycles at 95 °C for 5 s and 60 °C for 30 s. Melting curve analysis was performed to verify primer specificity. Relative mRNA expression was calculated using the $2^{-\Delta \Delta Ct}$ method. For each sample, ΔCt was calculated relative to *GAPDH*. The mean ΔCt of the control group was used as the calibrator to calculate ΔΔCt values for all individual samples, including those in the control group. Relative mRNA expression was then expressed as $2^{-\Delta \Delta Ct}$, allowing the variability among individual control samples to be displayed.

### 2.6. Enzyme-Linked Immunosorbent Assay (ELISA)

PIK3CA protein and 8-OHdG levels were measured using commercial ELISA kits according to the manufacturer’s instructions. PIK3CA protein levels were quantified in HUVEC lysates using the human PIK3CA ELISA kit (Elabscience, Houston, TX, USA), whereas 8-OHdG levels were measured in cell culture supernatants using the human 8-OHdG ELISA kit (Elabscience, Houston, TX, USA). Absorbance was measured at 450 nm using a microplate reader, and concentrations were calculated based on standard curves generated from known standards. PIK3CA protein levels were normalized to the total protein concentration of each cell lysate and expressed as ng/mg protein. 8-OHdG concentrations in the culture supernatants were expressed as ng/mL.

### 2.7. Measurement of Intracellular ROS Levels

Intracellular ROS levels were measured using the dichlorodihydrofluorescein diacetate (DCFH-DA) fluorometric assay (Elabscience, Houston, TX, USA) according to the manufacturer’s instructions. Briefly, HUVECs were incubated with 10 μM DCFH-DA working solution at 37 °C in the dark for 30 min. Cells were then harvested by trypsinization, centrifuged at 1000× *g* for 10 min, and washed twice with the assay buffer. The cell pellet was resuspended in assay buffer, transferred to a black 96-well microplate, and fluorescence intensity was measured using a fluorescence microplate reader at excitation and emission wavelengths of 500 and 525 nm, respectively. Background fluorescence from the assay buffer was subtracted from all sample readings prior to analysis, and intracellular ROS levels were expressed as relative fluorescence units (RFU).

### 2.8. Measurement of Total SOD Activity

Total SOD activity was measured using a total SOD activity assay kit (Elabscience Biotechnology Inc., Houston, TX, USA) based on the water-soluble tetrazolium salt-1 (WST-1) method according to the manufacturer’s instructions. The assay is based on the inhibition of WST-1 reduction by SOD, whereby superoxide anions generated in the reaction system reduce WST-1 to a water-soluble formazan dye that is quantified colorimetrically at 450 nm. Briefly, 20 μL of HUVEC lysate and 20 μL of enzyme working solution were added to sample wells, while the control wells contained 20 μL of double-distilled water and 20 μL of enzyme working solution. Blank control wells contained 20 μL of double-distilled water and 20 μL of enzyme diluent. Subsequently, 200 μL of substrate application solution was added to each well, and the plate was incubated at 37 °C for 20 min. Absorbance was measured at 450 nm using a microplate reader. The SOD inhibition ratio was calculated as:

$$\mathrm{Inhibition}\;(\%)=\frac{\left(A_{Control}-A_{BlankControl}\right)-\left(A_{Sample}-A_{BlankControl}\right)}{\left(A_{Control}-A_{BlankControl}\right)}\times 100$$

*A*_Control_, *A*_BlankControl_, and *A*_Sample_ represent the absorbance values of the control, blank control, and sample wells, respectively. One unit of SOD activity was defined as the amount of enzyme required to produce a 50% inhibition ratio in the reaction system. SOD activity was calculated according to the manufacturer’s instructions, normalized to the total protein concentration of each sample, and expressed as units per milligram protein (U/mg protein).

### 2.9. Statistical Analysis

Statistical analyses were performed using GraphPad Prism 9.0 (GraphPad Software Inc., La Jolla, CA, USA). Data are presented as mean ± standard error of the mean (SEM), with individual biological replicate values shown in the figures. Data distribution was assessed using the Shapiro–Wilk test together with inspection of the individual data points. As no significant departures from normality or obvious extreme outliers were identified, comparisons between two independent groups were performed using an unpaired Student’s *t*-test. A *p*-value < 0.05 was considered statistically significant. Biological replicates were derived from independent primary HUVEC preparations obtained from different human umbilical cords; thus, *n* = 4–6 represents 4–6 independent donors. The exact number of biological replicates (*n*) for each experiment is indicated in the corresponding figure legends.

## 3. Results

### 3.1. Bioinformatic Analysis Identifies PIK3CA as a Predicted Target of Putative Novel miR-1133

**Figure 1:**
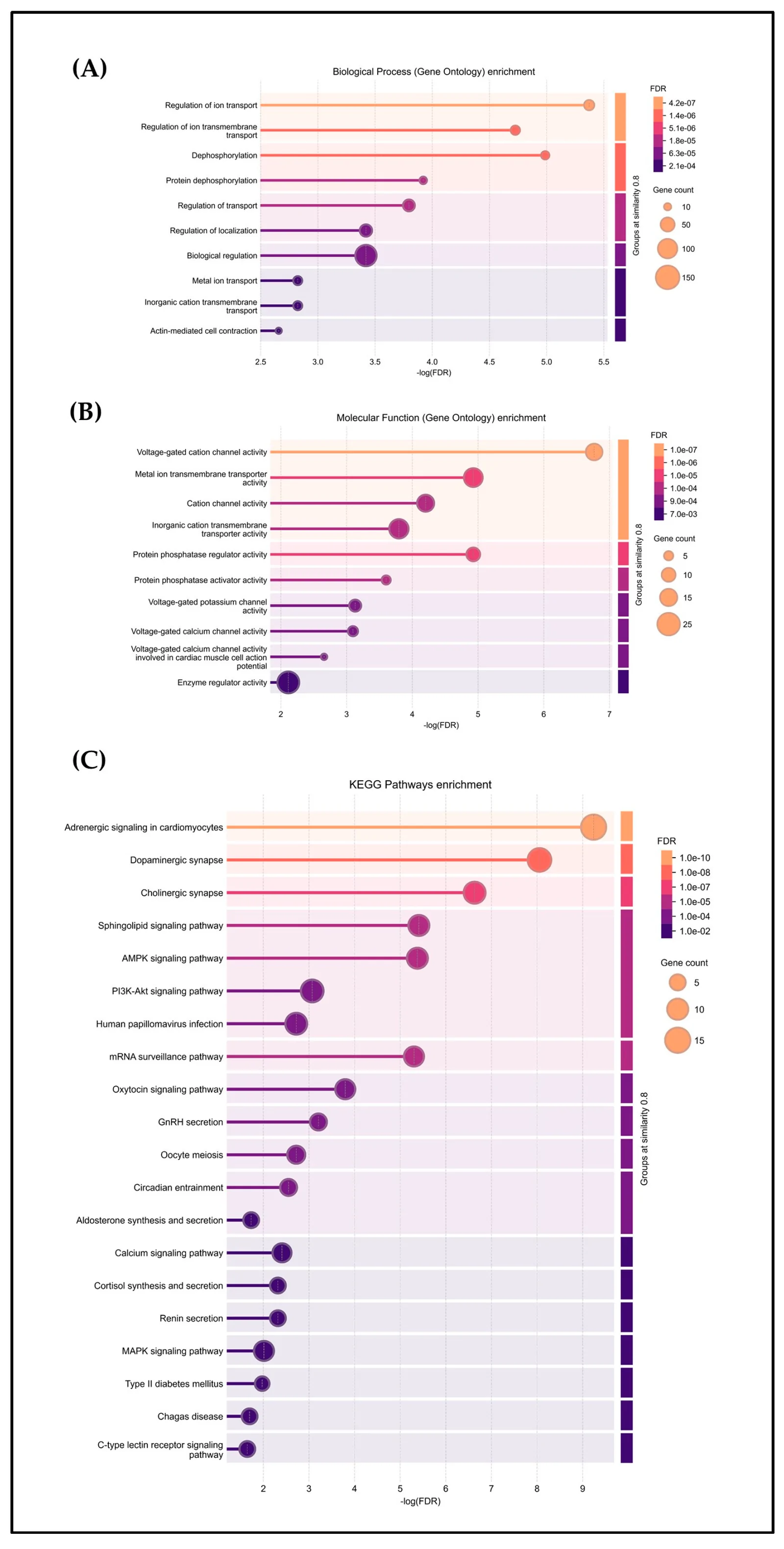
Functional enrichment analysis of predicted target genes of putative novel miR-1133. (**A**) Gene Ontology (GO) enrichment analysis of biological processes associated with the predicted target genes of putative novel miR-1133. (**B**) GO enrichment analysis of molecular functions associated with the predicted target genes. (**C**) Kyoto Encyclopedia of Genes and Genomes (KEGG) pathway enrichment analysis showing significantly enriched signaling pathways associated with the predicted target genes. The x-axis represents −log(FDR), indicating the level of statistical significance of enrichment, while the circle size corresponds to the number of genes associated with each GO term or KEGG pathway. The color gradient represents the false discovery rate (FDR), with darker colors indicating greater statistical significance.

A total of 132 predicted target genes of putative novel miR-1133 were identified from our previous RNA-sequencing study [19]. Gene Ontology (GO) enrichment analysis revealed that the predicted target genes were associated with several biological processes, including regulation of ion transport, regulation of ion transmembrane transport, protein dephosphorylation and biological regulation (Figure 1A). The enriched molecular functions included metal ion transmembrane transporter activity, cation channel activity, protein phosphatase regulator activity and voltage-gated calcium channel activity (Figure 1B). KEGG pathway analysis identified several significantly enriched pathways, including the PI3K/Akt signaling pathway, 5’ AMP-activated protein kinase (AMPK) signaling pathway, mitogen-activated protein kinase (MAPK) signaling pathway and type II diabetes mellitus pathway (Figure 1C).

**Figure 2:**
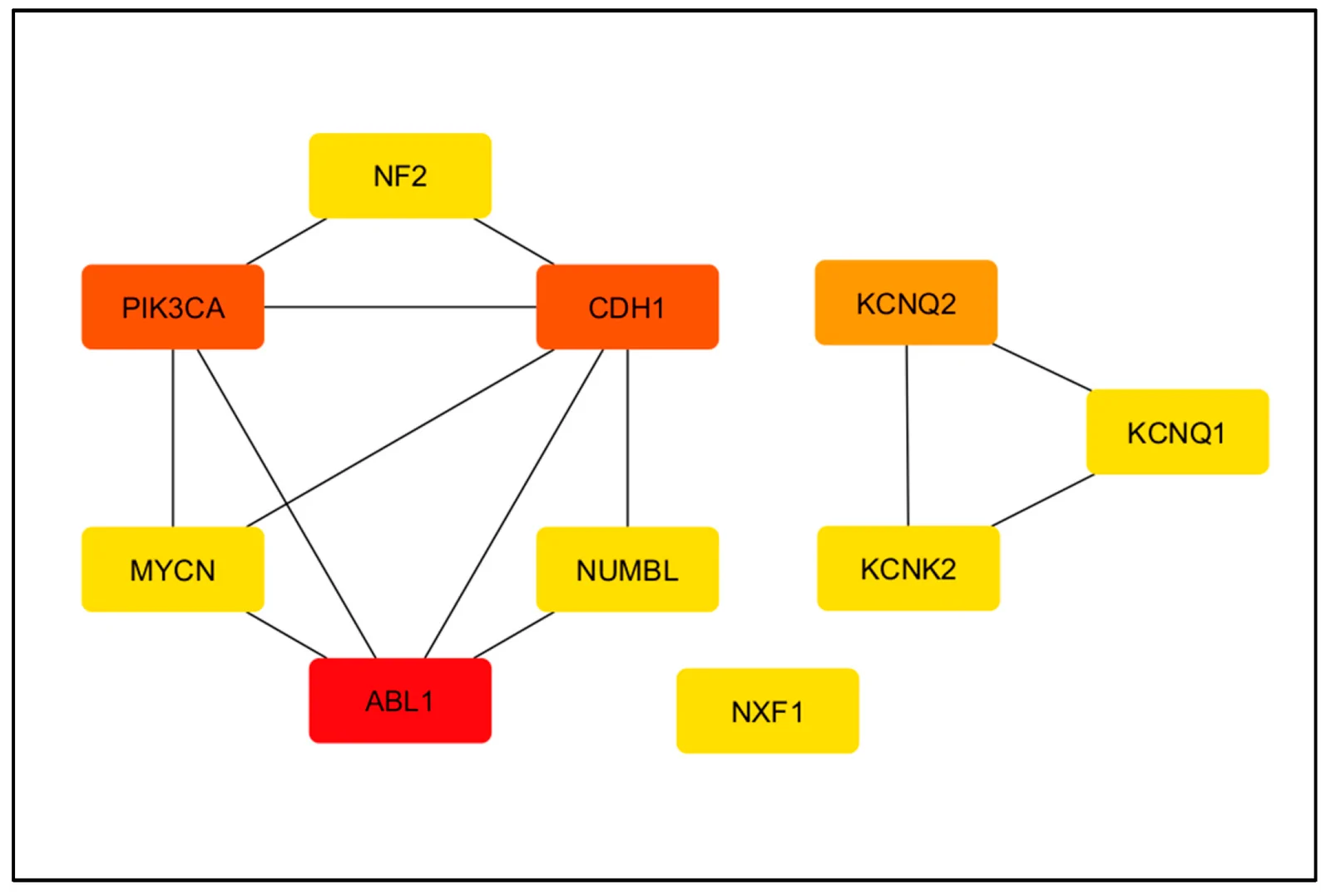
Protein–protein interaction (PPI) network analysis of the predicted target genes of putative novel miR-1133. The top 10 hub genes were identified using the CytoHubba plugin in Cytoscape based on degree centrality analysis. Node colors range from red to yellow, representing genes ranked from the highest to the lowest interaction degree, respectively. Among the identified hub genes, *ABL1, PIK3CA* and *CDH1* exhibited the highest interaction degrees within the network. *ABL1*, ABL proto-oncogene 1, non-receptor tyrosine kinase; *CDH1*, cadherin 1; *KCNK2*, potassium channel, subfamily K, member 2; *KCNQ1*, voltage-gated potassium channel, subfamily Q, member 1; *KCNQ2*, voltage-gated potassium channel, subfamily Q, member 2; *MYCN*, MYCN proto-oncogene, bHLH transcription factor; *NF2*, neurofibromin 2; *NUMBL*, NUMB-like; NXF1, nuclear RNA export factor 1; *PIK3CA*, phosphatidylinositol-4,5-bisphosphate 3-kinase catalytic subunit alpha.

To identify key regulatory genes, a PPI network was constructed using Cytoscape. Among the top ten hub genes identified, ABL proto-oncogene 1, non-receptor tyrosine kinase (*ABL1*), *PIK3CA* and cadherin 1 (*CDH1*) exhibited the highest interaction degrees (Figure 2). Based on its high interaction degree in the PPI network, together with the significant enrichment of the PI3K/Akt signaling pathway, *PIK3CA* was selected for further experimental investigation. The established involvement of the PI3K/Akt pathway in oxidative stress regulation and diabetic endothelial dysfunction [20,21,22] provided the rationale for prioritizing *PIK3CA* for further investigation under hyperglycemic conditions.

### 3.2. Effects of Hyperglycemia and Putative Novel miR-1133 Inhibitor Transfection on PIK3CA Expression in HUVECs

**Figure 3:**
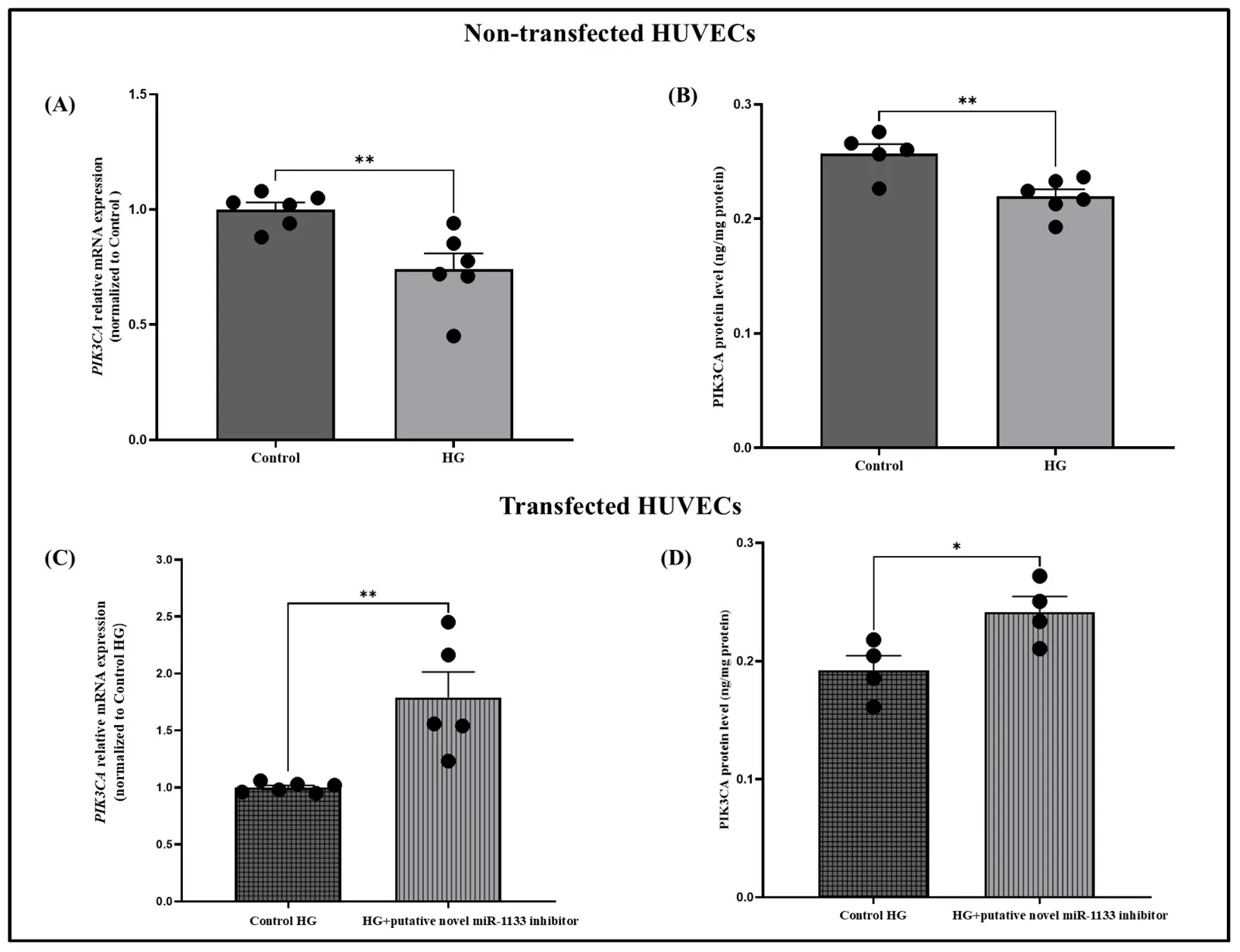
Effects of hyperglycemia and putative novel miR-1133 inhibitor transfection on phosphatidylinositol-4,5-bisphosphate 3-kinase catalytic subunit alpha (PIK3CA) expression. Panels A and B correspond to non-transfected HUVECs and show the effects of hyperglycemia on (**A**) *PIK3CA* mRNA expression and (**B**) PIK3CA protein levels in control and hyperglycemia (HG)-treated HUVECs. Panels C and D represent transfected HUVECs and show the effects of putative novel miR-1133 inhibitor transfection on (**C**) *PIK3CA* mRNA expression and (**D**) PIK3CA protein levels in the transfection control (control HG) and HG + putative novel miR-1133 inhibitor groups. The transfection control group consisted of HG-induced HUVECs transfected with Lipofectamine^®^ only. Data are presented as mean ± SEM (*n* = 4–6); * *p* < 0.05, ** *p* < 0.01.

Based on the bioinformatic identification of *PIK3CA* as a predicted hub gene, we next examined whether hyperglycemia and transfection with putative novel miR-1133 inhibitor were associated with changes in *PIK3CA* expression in HUVECs. Hyperglycemia (HG) significantly reduced *PIK3CA* mRNA expression (*p* < 0.01) and PIK3CA protein levels compared with the control cells (*p* < 0.01) (Figure 3A,B). To evaluate the effect of putative novel miR-1133 inhibitor transfection, HG-induced HUVECs were transfected with a putative novel miR-1133 inhibitor during continued hyperglycemic exposure. Putative novel miR-1133 inhibitor transfection significantly increased *PIK3CA* mRNA expression (*p* < 0.01) and PIK3CA protein levels (*p* < 0.05) compared with the transfection control group (Figure 3C,D).

### 3.3. Effects of Hyperglycemia and Putative Novel miR-1133 Inhibitor Transfection on Oxidative Stress Markers in HUVECs

**Figure 4:**
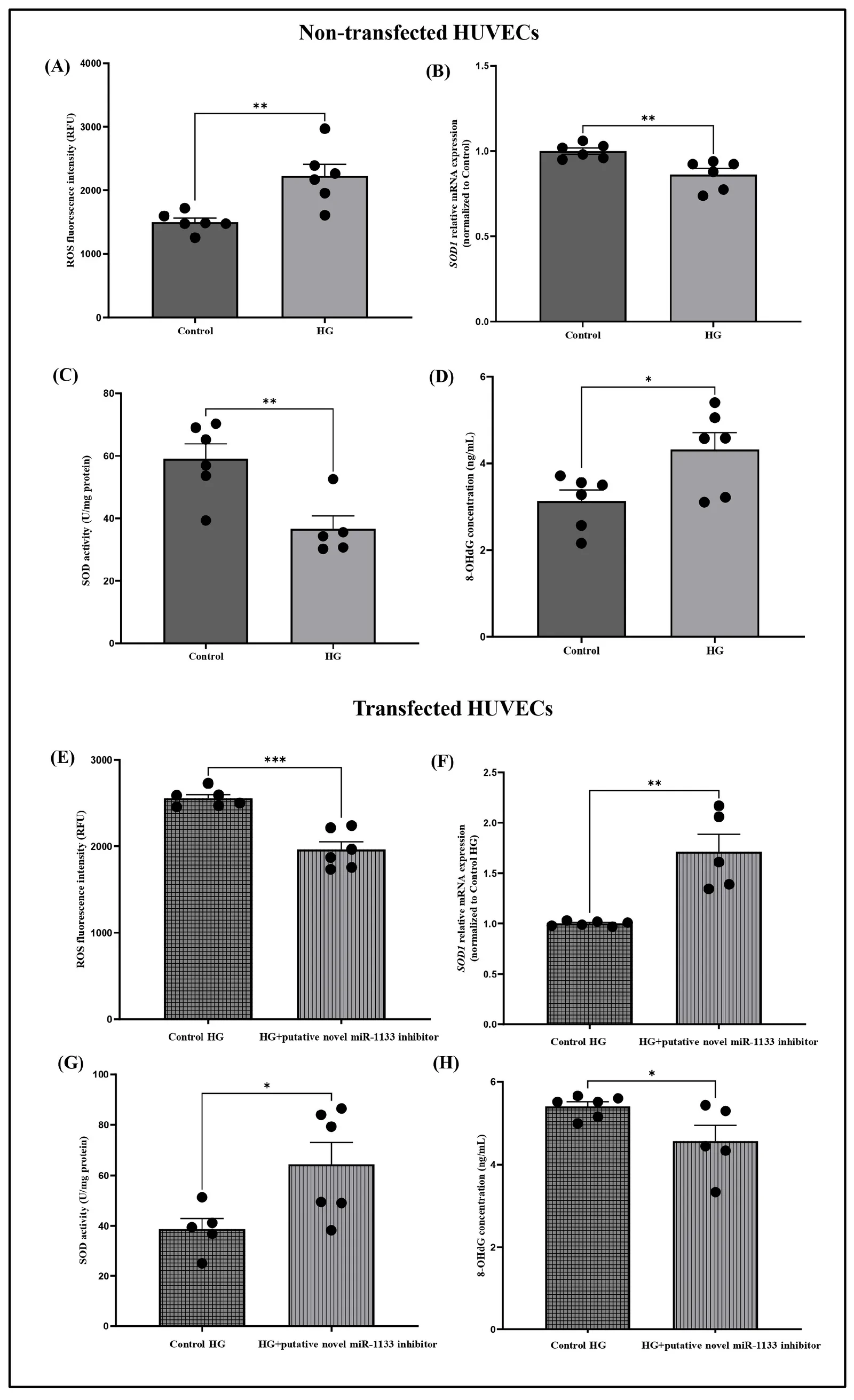
Effects of hyperglycemia and putative novel miR-1133 inhibitor transfection on oxidative stress markers in HUVECs. Panels A–D correspond to non-transfected HUVECs and represent the effects of hyperglycemia on (**A**) intracellular reactive oxygen species (ROS) levels, (**B**) superoxide dismutase 1 (*SOD1*) mRNA expression, (**C**) SOD activity, and (**D**) 8-hydroxy-2′-deoxyguanosine (8-OHdG) levels in control and hyperglycemia (HG)-treated HUVECs. Panels E–H represent transfected HUVECs and show the effects of putative novel miR-1133 inhibitor transfection on (**E**) intracellular ROS levels, (**F**) *SOD1* mRNA expression, (**G**) total SOD activity, and (**H**) 8-OHdG levels in the transfection control (control HG) and HG + putative novel miR-1133 inhibitor groups. The transfection control group consisted of HG-induced HUVECs transfected with Lipofectamine^®^ only. Data are presented as mean ± SEM (*n* = 5–6); * *p* < 0.05, ** *p* < 0.01 and *** *p* < 0.001.

The effects of hyperglycemia and putative novel miR-1133 inhibitor transfection on oxidative stress were evaluated by measuring intracellular ROS levels, *SOD1* mRNA expression, total SOD activity, and 8-OHdG levels (Figure 4). Hyperglycemia significantly increased intracellular ROS (*p* < 0.01) and 8-OHdG (*p* < 0.05) levels while reducing *SOD1* mRNA expression (*p* < 0.01) and total SOD activity (*p* < 0.01) compared with the control group (Figure 4A–D). To determine the effects of putative novel miR-1133 inhibitor transfection on oxidative stress, HG-induced HUVECs were transfected with a putative novel miR-1133 inhibitor. Putative novel miR-1133 inhibitor transfection significantly reduced intracellular ROS (*p* < 0.001) and 8-OHdG levels (*p* < 0.05) while significantly increasing *SOD1* mRNA expression (*p* < 0.01) and total SOD activity (*p* < 0.05) compared with the transfection control group (Figure 4E–H).

## 4. Discussion

Building upon our previous RNA sequencing study, in which putative novel miR-1133 was predicted using the miRDeep2 algorithm and identified as the most significantly upregulated miRNA in hyperglycemia-induced HUVECs, with 132 potential target genes subsequently predicted using miRDB [19], the present study explored the potential involvement of putative novel miR-1133 in oxidative stress in hyperglycemia-induced endothelial cells through bioinformatic and experimental analyses. The findings demonstrated that hyperglycemia reduced *PIK3CA* expression and increased oxidative stress, whereas transfection with putative novel miR-1133 inhibitor was associated with increased *PIK3CA* expression, reduced ROS and 8-OHdG levels, and enhanced antioxidant defense through increased *SOD1* expression and total SOD activity. Together, these findings suggest an association between putative novel miR-1133 inhibitor transfection, *PIK3CA* expression, and oxidative stress under hyperglycemic conditions. However, they do not establish a direct regulatory relationship between putative novel miR-1133 and *PIK3CA*.

Bioinformatic analyses identified *PIK3CA* as one of the major hub genes among the predicted targets of putative novel miR-1133. *PIK3CA* encodes the catalytic α-subunit of PI3K and is essential for PI3K enzymatic activity [27]. Functional enrichment analyses further identified significant enrichment of pathways associated with diabetic endothelial dysfunction, including the PI3K/Akt, AMPK, and MAPK signaling pathways. Among these, the PI3K/Akt pathway was of particular interest because of its established role in endothelial homeostasis and oxidative stress regulation [28]. Activation of the PI3K/Akt pathway promotes endothelial survival, enhances NO bioavailability through endothelial nitric oxide synthase activation, and protects against oxidative injury. Dysregulation of this pathway has been implicated in endothelial dysfunction and vascular complications associated with diabetes [29].

In the present study, hyperglycemia significantly reduced both *PIK3CA* mRNA and protein expression in HUVECs. These findings are consistent with previous reports demonstrating impaired PI3K/Akt signaling under hyperglycemic conditions. Hyperglycemia has been shown to suppress PI3K signaling, resulting in impaired endothelial function and reduced cellular resistance to oxidative injury [22,30]. Given that *PIK3CA* encodes the catalytic alpha subunit responsible for PI3K activity [27], the observed reduction in *PIK3CA* expression may be relevant to impaired endothelial protective signaling during hyperglycemia, although downstream PI3K/Akt signaling was not assessed in the present study.

To further investigate the association between putative novel miR-1133 and *PIK3CA*, hyperglycemia-induced HUVECs were transfected with a putative novel miR-1133 inhibitor. Putative novel miR-1133 inhibitor transfection significantly increased both *PIK3CA* mRNA and protein expression. Together with the bioinformatic prediction, these findings indicate an association between putative novel miR-1133 inhibitor transfection and increased *PIK3CA* expression under hyperglycemic conditions. However, the present study does not establish a direct regulatory relationship between putative novel miR-1133 and *PIK3CA*, as direct target binding was not evaluated. Therefore, *PIK3CA* should be considered a predicted downstream target of putative novel miR-1133 that warrants further validation.

Oxidative stress is a major contributor to endothelial dysfunction in diabetes and is characterized by excessive ROS production and impaired antioxidant defense mechanisms [30]. Consistent with previous studies, hyperglycemia significantly increased intracellular ROS levels while reducing *SOD1* expression and total SOD activity [31]. These changes were accompanied by increased 8-OHdG levels, consistent with oxidative DNA damage. Elevated glucose concentrations promote ROS generation through multiple mechanisms, including activation of protein kinase C and NADPH oxidase, leading to oxidative injury and endothelial dysfunction [32].

In the present study, putative novel miR-1133 inhibitor transfection was associated with reduced oxidative stress in hyperglycemia-induced HUVECs. Specifically, putative novel miR-1133 inhibitor transfection was associated with reduced intracellular ROS and 8-OHdG levels while increasing *SOD1* expression and total SOD activity. Collectively, these findings indicate an association between putative novel miR-1133 inhibitor transfection and attenuation of oxidative stress under hyperglycemic conditions. The observed increase in *SOD1* expression and total SOD activity may be biologically relevant because SOD serves as a first-line antioxidant enzyme that protects endothelial cells against superoxide-mediated oxidative damage [33].

A potential mechanistic link between putative novel miR-1133, *PIK3CA* expression and oxidative stress may involve the PI3K/Akt signaling pathway. Previous studies have shown that activation of PI3K/Akt signaling promotes antioxidant responses through downstream mediators such as nuclear factor erythroid 2–related factor 2 *(NRF2)*, which regulates the expression of antioxidant enzymes including SOD [34]. Thus, the increased *PIK3CA* expression and attenuation of oxidative stress observed following putative novel miR-1133 inhibitor transfection raise the possibility that PI3K/Akt signaling may contribute to the observed redox changes. However, Akt activation was not assessed in the present study, and no causal relationship between increased *PIK3CA* expression and attenuation of oxidative stress can be established from the current data. Therefore, the potential involvement of PI3K/Akt signaling remains a hypothesis that requires direct experimental validation.

**Figure 5:**
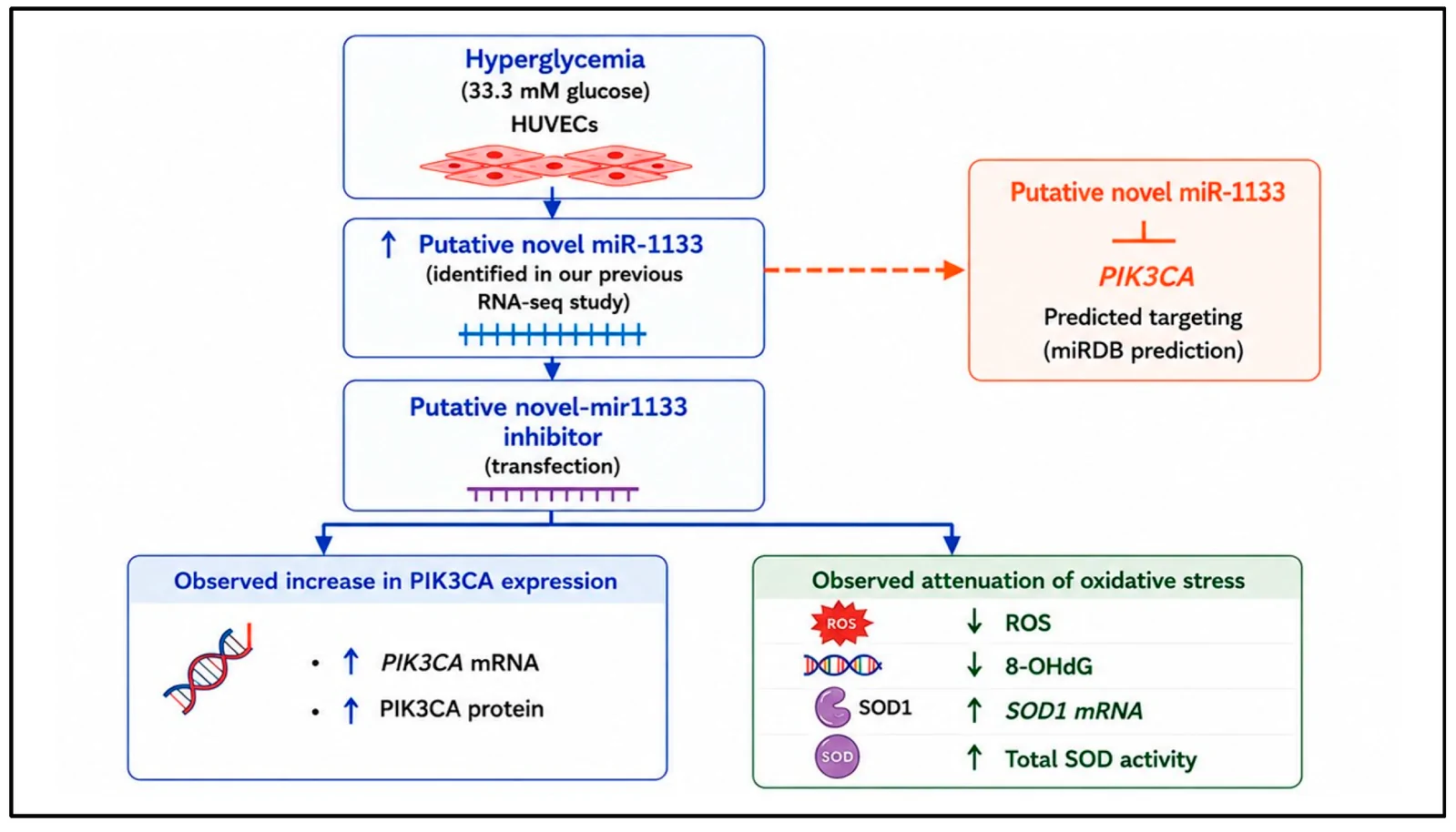
Summary of the bioinformatic and experimental findings on the association between putative novel miR-1133, *PIK3CA* expression, and oxidative stress in hyperglycemia-induced endothelial cells. Hyperglycemia increased the expression of putative novel miR-1133 in HUVECs, as identified in our previous RNA-sequencing study. Bioinformatic analysis predicted *PIK3CA* as a potential target of putative novel miR-1133. Transfection of HUVECs with putative novel miR-1133 inhibitor was associated with increased *PIK3CA* mRNA and PIK3CA protein expression, together with reduced intracellular reactive oxygen species (ROS) and 8-hydroxy-2′-deoxyguanosine (8-OHdG) levels and increased *SOD1* mRNA expression and total superoxide dismutase (SOD) activity. Solid arrows indicate experimentally supported findings, whereas dashed arrows indicate bioinformatically predicted interactions. The observed association between increased *PIK3CA* expression and attenuation of oxidative stress does not establish a causal relationship or involvement of a specific downstream signaling pathway.

Figure 5 summarizes the bioinformatic and experimental findings of the present study. The predicted interaction between putative novel miR-1133 and *PIK3CA* is based on bioinformatic analysis, whereas increased *PIK3CA* expression and attenuation of oxidative stress following putative novel miR-1133 inhibitor transfection represent experimentally observed associations. The present findings do not establish a causal relationship between increased *PIK3CA* expression and attenuation of oxidative stress or demonstrate the involvement of a specific downstream signaling pathway.

Nevertheless, several limitations of this study should be acknowledged. The study was conducted using an in vitro HUVEC model, which may not fully recapitulate the complexity of diabetic vascular disease in vivo. Therefore, the findings should be interpreted as exploratory cellular observations and require validation in animal models and human tissues. Although the miRNA inhibitor transfection protocol was validated using hsa-let-7c inhibitor as a positive control, inhibition of endogenous putative novel miR-1133 was not directly quantified. Therefore, the inhibitory efficacy of the putative novel miR-1133 inhibitor remains to be directly established. Furthermore, a non-targeting miRNA inhibitor was not included as a control; therefore, potential sequence-independent effects associated with inhibitor oligonucleotide transfection cannot be excluded. Although bioinformatic analyses and expression studies suggested an association between putative novel miR-1133 and *PIK3CA*, direct target binding was not evaluated. Future studies employing luciferase reporter assays with mutant constructs are required to determine whether a direct interaction exists, while *PIK3CA* gain- and loss-of-function and rescue experiments are needed to determine whether the observed changes in oxidative stress are causally dependent on *PIK3CA*. Consequently, the inverse relationship observed between putative novel miR-1133 inhibitor transfection and *PIK3CA* expression should be interpreted as evidence of an association rather than definitive evidence of direct post-transcriptional regulation. Furthermore, only one predicted target gene of putative novel miR-1133 was investigated despite the identification of 132 potential target genes. Future studies should evaluate additional target genes and downstream signaling pathways to further elucidate the biological roles of putative novel miR-1133 under diabetic or hyperglycemic conditions.

Additionally, an osmotic control was not included to distinguish glucose-specific effects from nonspecific osmotic stress, and future studies incorporating appropriate osmotic controls would strengthen the interpretation of the findings. Functional assays evaluating endothelial cell viability, apoptosis, migration, tube formation, or inflammatory responses were also not performed and would provide complementary evidence of the biological consequences of putative novel miR-1133 inhibitor transfection in endothelial cells. Finally, RT-qPCR normalization was performed using a single reference gene (*GAPDH*), and primer amplification efficiency was not experimentally determined. The inclusion of multiple validated reference genes and assessment of primer amplification efficiency would further strengthen the robustness and reliability of RT-qPCR analysis in future studies.

## 5. Conclusions

The present study explored the potential involvement of putative novel miR-1133 in oxidative stress under hyperglycemic conditions in endothelial cells. Transfection with putative novel miR-1133 inhibitor was associated with reduced intracellular ROS and 8-OHdG levels together with increased *SOD1* expression and total SOD activity. Bioinformatic analysis identified *PIK3CA* as a predicted downstream target of putative novel miR-1133, and increased *PIK3CA* mRNA and PIK3CA protein expression was observed following transfection with putative novel miR-1133 inhibitor. Collectively, these findings support an association between putative novel miR-1133 inhibitor transfection, *PIK3CA* expression, and oxidative stress under hyperglycemic conditions. Further studies are required to validate the direct interaction between putative novel miR-1133 and *PIK3CA* and to determine the relevance of these findings in vivo.

## Data Availability

The data presented in this study are available from the corresponding author upon reasonable request.

## Supplementary Materials

The following supporting information can be downloaded at https://www.mdpi.com/article/10.3390/biomedicines14092038/s1, Supplementary Table S1: list of target genes for putative novel miR-1133; Supplementary Figure S1. Validation of the miRNA inhibitor transfection protocol in hyperglycemia-induced HUVECs.

## Abbreviations

The following abbreviations are used in this manuscript:

3′UTR	3′ untranslated region
8-OHdG	8-hydroxy-2′-deoxyguanosine
ABL1	ABL proto-oncogene 1, non-receptor tyrosine kinase
Akt	Protein kinase B
AMPK	5’ AMP-activated protein kinase
CDH1	Cadherin 1
cDNA	Complementary DNA
DM	Diabetes mellitus
DNA	Deoxyribonucleic acid
ELISA	Enzyme-linked immunosorbent assay
GO	Gene Ontology
HG	Hyperglycemia
HUVECs	Human umbilical vein endothelial cells
KEGG	Kyoto Encyclopedia of Genes and Genomes
MAPK	Mitogen-activated protein kinase
miRNA	MicroRNA
mRNA	Messenger RNA
NADPH	Nicotinamide adenine dinucleotide phosphate
NO	Nitric oxide
NRF2	Nuclear factor erythroid 2–related factor 2
PI3K	Phosphatidylinositol 3-kinase
PIK3CA	Phosphatidylinositol-4,5-bisphosphate 3-kinase catalytic subunit alpha
PKC	Protein kinase C
PPI	Protein–protein interaction
qPCR	Quantitative polymerase chain reaction
ROS	Reactive oxygen species
SEM	Standard error of the mean
SOD	Superoxide dismutase
STRING	Search Tool for the Retrieval of Interacting Genes/Proteins
ΔΔCT	Double delta threshold cycle
