# Supplementary material for: Putative Novel miR-1133 Is Associated with Oxidative Stress in Hyperglycemia-Induced Endothelial Cells
Source: Biomedicines. 2026 Sep 10;14(9):2038. doi: 10.3390/biomedicines14092038 (PMC13604605; doi:10.3390/biomedicines14092038)
Supplement: Supplementary file 1 [file biomedicines-14-02038-s001.zip › biomedicines-4519530-supplementary.pdf]

Supplementary Table S1: Predicted target genes of putative novel miR-1133

Target Score	Gene Symbol	Gene Description
94	MEX3C	mex-3 RNA binding family member C
91	NF2	neurofibromin 2
90	FBXO33	F-box protein 33
87	VKORC1L1	vitamin K epoxide reductase complex subunit 1 like 1
85	APOBEC3D	apolipoprotein B mRNA editing enzyme catalytic subunit 3D
84	DUSP18	dual specificity phosphatase 18
82	AHI1	Abelson helper integration site 1
81	ACP6	acid phosphatase 6, lysophosphatidic
80	IFT122	intraflagellar transport 122
78	AKAP13	A-kinase anchoring protein 13
78	ARRDC3	arrestin domain containing 3
78	SZRD1	SUZ RNA binding domain containing 1
78	NKAIN1	sodium/potassium transporting ATPase interacting 1
77	MAP2K4	mitogen-activated protein kinase kinase 4
76	CNTN1	contactin 1
76	UBE2O	ubiquitin conjugating enzyme E2 O
74	RFT1	RFT1 homolog
74	KCNK2	potassium two pore domain channel subfamily K member 2
73	PADI2	peptidyl arginine deiminase 2
73	PHF21A	PHD finger protein 21A
73	H6PD	hexose-6-phosphate dehydrogenase/glucose 1-dehydrogenase
72	RIMS4	regulating synaptic membrane exocytosis 4
72	QKI	QKI, KH domain containing RNA binding
72	TMC1	transmembrane channel like 1
72	YTHDF1	YTH N6-methyladenosine RNA binding protein 1
72	SLX4	SLX4 structure-specific endonuclease subunit
71	NUTF2	nuclear transport factor 2
70	SEC63	SEC63 homolog, protein translocation regulator
70	IQSEC3	IQ motif and Sec7 domain 3
70	NXF1	nuclear RNA export factor 1
69	BPGM	bisphosphoglycerate mutase
69	NRP1	neuropilin 1
68	SNX27	sorting nexin family member 27
68	ACAP3	ArfGAP with coiled-coil, ankyrin repeat and PH domains 3
68	CACNA1I	calcium voltage-gated channel subunit alpha1 I
68	SPRN	shadow of prion protein
67	CDH1	cadherin 1
67	SPEF1	sperm flagellar 1
67	LGI3	leucine rich repeat LGI family member 3
65	TRABD2B	TraB domain containing 2B
65	EXTL3	exostosin like glycosyltransferase 3
65	CNNM3	cyclin and CBS domain divalent metal cation transport mediator 3
65	KDM2B	lysine demethylase 2B
64	MOB3A	MOB kinase activator 3A
64	THRB	thyroid hormone receptor beta
64	FBXL20	F-box and leucine rich repeat protein 20
64	PRND	prion like protein doppel

64	ALK	ALK receptor tyrosine kinase
63	VSIG4	V-set and immunoglobulin domain containing 4
63	PPP3CB	protein phosphatase 3 catalytic subunit beta
63	INMT	indolethylamine N-methyltransferase
62	ALG12	ALG12, alpha-1,6-mannosyltransferase
62	SHISA7	shisa family member 7
61	FMO5	flavin containing monooxygenase 5
61	ERLIN1	ER lipid raft associated 1
61	LRFN1	leucine rich repeat and fibronectin type III domain containing 1
61	MMP24	matrix metallopeptidase 24
61	RLIM	ring finger protein, LIM domain interacting
61	FCRL3	Fc receptor like 3
61	TNC	tenascin C
60	ARNT2	aryl hydrocarbon receptor nuclear translocator 2
59	HOXA13	homeobox A13
59	SMAD6	SMAD family member 6
59	NDOR1	NADPH dependent diflavin oxidoreductase 1
59	GRHL2	grainyhead like transcription factor 2
59	ADM5	adrenomedullin 5 (putative)
59	GPR132	G protein-coupled receptor 132
58	SNX15	sorting nexin 15
58	SLC23A2	solute carrier family 23 member 2
58	TRPV3	transient receptor potential cation channel subfamily V member 3
58	RAB11FIP2	RAB11 family interacting protein 2
57	ARMC5	armadillo repeat containing 5
57	PCBP4	poly(rC) binding protein 4
57	GINS3	GINS complex subunit 3
57	SLC35F6	solute carrier family 35 member F6
57	PIK3CA	phosphatidylinositol-4,5-bisphosphate 3-kinase catalytic subunit alpha
56	KCNQ1	potassium voltage-gated channel subfamily Q member 1
56	PTGES3L	prostaglandin E synthase 3 like
56	LRRC41	leucine rich repeat containing 41
56	CMKLR1	chemerin chemokine-like receptor 1
56	TVP23C	trans-golgi network vesicle protein 23 homolog C
56	MARCKSL1	MARCKS like 1
56	TBC1D24	TBC1 domain family member 24
56	MCIDAS	multiciliate differentiation and DNA synthesis associated cell cycle protein
56	PPM1A	protein phosphatase, Mg2+/Mn2+ dependent 1A
55	IGSF11	immunoglobulin superfamily member 11
55	PPP2R5B	protein phosphatase 2 regulatory subunit B'beta
55	NOS1	nitric oxide synthase 1
55	NACC1	nucleus accumbens associated 1
55	PODNL1	podocan like 1
54	DPYSL5	dihydropyrimidinase like 5
54	MYCN	MYCN proto-oncogene, bHLH transcription factor
54	SKIL	SKI like proto-oncogene
54	SH3BP2	SH3 domain binding protein 2
54	KCNB1	potassium voltage-gated channel subfamily B member 1
54	FRMD6	FERM domain containing 6
54	NUMBL	NUMB like, endocytic adaptor protein

53	LHPP	phospholysine phosphohistidine inorganic pyrophosphate phosphatase
53	CHRM1	cholinergic receptor muscarinic 1
53	PRR14L	proline rich 14 like
53	SDC3	syndecan 3
53	SLC30A5	solute carrier family 30 member 5
53	PACS1	phosphofurin acidic cluster sorting protein 1
53	CCL22	C-C motif chemokine ligand 22
53	NAT8L	N-acetyltransferase 8 like
53	NANP	N-acetylneuraminic acid phosphatase
52	MCUR1	mitochondrial calcium uniporter regulator 1
52	KHDC3L	KH domain containing 3 like, subcortical maternal complex member
52	SS18L1	SS18L1, nBAF chromatin remodeling complex subunit
52	SRD5A3	steroid 5 alpha-reductase 3
52	PPP2R5D	protein phosphatase 2 regulatory subunit B'delta
52	PML	promyelocytic leukemia
52	SLC30A6	solute carrier family 30 member 6
51	GYPC	glycophorin C (Gerbich blood group)
51	SAMD14	sterile alpha motif domain containing 14
51	APBB2	amyloid beta precursor protein binding family B member 2
51	ABL1	ABL proto-oncogene 1, non-receptor tyrosine kinase
51	CTDSP1	CTD small phosphatase 1
51	MAVS	mitochondrial antiviral signaling protein
51	TMPRSS4	transmembrane serine protease 4
51	ZNF426	zinc finger protein 426
51	TAF8	TATA-box binding protein associated factor 8
51	ATP8A1	ATPase phospholipid transporting 8A1
51	CYP4V2	cytochrome P450 family 4 subfamily V member 2
51	BBX	BBX, HMG-box containing
51	VSTM4	V-set and transmembrane domain containing 4
50	FGD3	FYVE, RhoGEF and PH domain containing 3
50	KCNQ2	potassium voltage-gated channel subfamily Q member 2
50	SPATA13	spermatogenesis associated 13
50	MEI1	meiotic double-stranded break formation protein 1
50	SRXN1	sulfiredoxin 1
50	CNOT6	CCR4-NOT transcription complex subunit 6

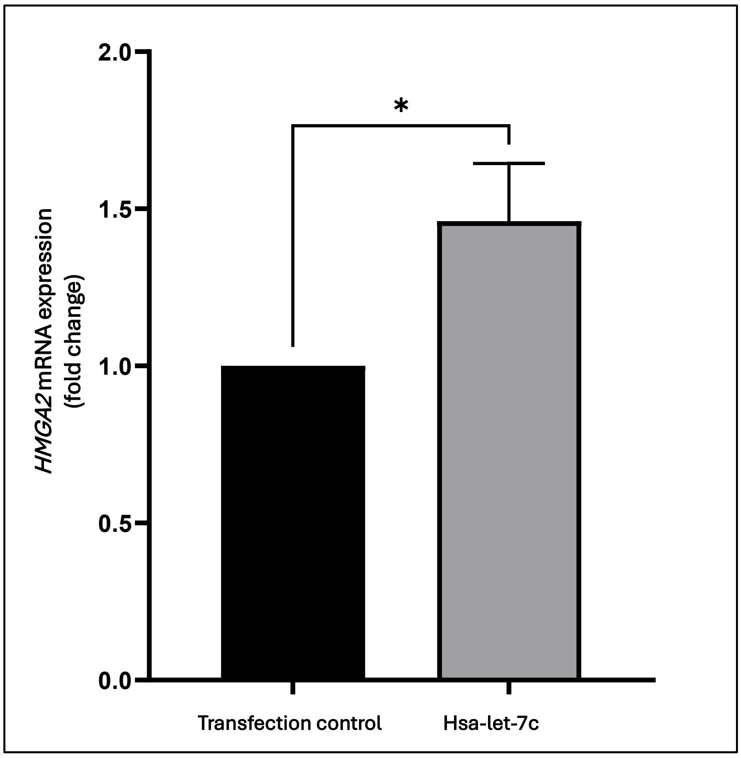

Supplementary Figure S1. Validation of the miRNA inhibitor transfection protocol in hyperglycemia-induced HUVECs. Hsa-let-7c inhibitor was used as a positive control miRNA inhibitor, and its functional activity was assessed by measuring the mRNA expression of its known target gene, *HMGA2*. Transfection with hsa-let-7c inhibitor significantly increased *HMGA2* mRNA expression compared with the transfection control (Lipofectamine alone). Data are presented as mean ± SEM, $n = 3$; $P < 0.05$ versus transfection control.
